# Use of Condoms: Clarifying the Message

**DOI:** 10.4103/0970-0218.66860

**Published:** 2010-04

**Authors:** JP Majra

**Affiliations:** Department of Community Medicine, K.S.Hegde Medical Academy, Mangalore, India E- mail: jpmajra@hotmail.com

Sir,

The emergence of human immunodeficiency virus (HIV) and identification of sexually transmitted infections (STIs) as a co-factor have revived the interest of the scientific community in condoms. Condom use in India has increased from 2.1% in 1992 – 1993 (NFHS-1) to 5.2% in 2005–2006 (NFHS-3) among males aged 15–54 years.[1] However, condoms are not 100% safe, but if used consistently and correctly, will reduce the risk of pregnancy and/or STIs significantly. The partners who consistently used condoms had a near zero risk of HIV, while inconsistent use carried considerable risk averaging to about 14–21% (an incidence of 4.8–5.4 per 100 persons/year)[2] Studies have also confirmed that consistent and correct use of condoms is the most important factor in preventing pregnancy. In general, the failure rate for perfect use (i.e., a condom used correctly at every act of intercourse) is approximately 3%, and for typical use (condoms not used for every act of intercourse) the failure rate is 12%. A large number of studies on condom breakage, report rates that vary from less than 1% to more than 10%.[2] Although many people mistakenly assume that all men know how to correctly use condoms, incorrect use is common and is a major cause of condom failure. A survey on condom use showed that 42% of the men surveyed did not use a condom from the start and/or to completion of penetrative sex, 23% did not leave a space at the receptacle tip and 81% did not use a water-based lubricant.[3] To enhance the potential efficacy of condoms the Ministry of Health and Family Welfare, Government of India, has issued National Guidelines on condom and its proper usage technique.[4] Instruction number two under the heading ‘General instructions for condom use’ on page number 77, states ‘because it (condom) is made from polyurethane, you can use oil-based lubricants with the condom’. Sir, this instruction is in contradiction to instructions given by the WHO that states, ‘Do not use oil-based lubricants’.[5] Using oil-based lubricants can weaken the latex, causing the condom to break. Guidelines on condom and its proper usage technique (GoI) further mentions on page number 78 that most male condoms are made of latex, which are of two types: Non-lubricated and lubricated. In India, where most of the condom users (73.3%) use either free, socially marketed or condoms of unknown brand (which may require separate/additional lubrication),[1] any decrease in the potential efficacy of condoms as a result of oil-based lubricants may be a self-fulfilling prophecy, because condoms may be used less consistently by those who do not believe them to be effective. The issue deserves to be urgently discussed and clarified.
